# C-reactive protein concentration as a risk predictor of mortality in intensive care unit: a multicenter, prospective, observational study

**DOI:** 10.1186/s12871-020-01207-3

**Published:** 2020-11-23

**Authors:** Rong Qu, Linhui Hu, Yun Ling, Yating Hou, Heng Fang, Huidan Zhang, Silin Liang, Zhimei He, Miaoxian Fang, Jiaxin Li, Xu Li, Chunbo Chen

**Affiliations:** 1 The Second School of Clinical Medicine, Southern Medical University, Guangzhou, 510515 Guangdong China; 2 Department of Critical Care Medicine, Huizhou Municipal Central Hospital, 41 North E’ling Road, Huizhou, 516001 Guangdong China; 3 Department of Critical Care Medicine, Maoming People’s Hospital, 101 Weimin Road, Maoming, 525000 Guangdong China; 4 Clinical Research Center, Maoming People’s Hospital, 101 Weimin Road, Maoming, 525000 Guangdong China; 5 Department of Intensive Care Unit of Cardiovascular Surgery, Guangdong Cardiovascular Institute, Guangdong Provincial People’s Hospital, Guangdong Academy of Medical Sciences, 96 Dongchuan Road, Guangzhou, 510080 Guangdong China; 6 Department of Critical Care Medicine, Guangdong Provincial People’s Hospital, Guangdong Academy of Medical Sciences, 106 Zhongshan Er Road, Guangzhou, 510080 Guangdong China; 7 State Key Laboratory of Organ Failure Research, Guangdong Provincial Key Laboratory of Viral Hepatitis Research, Department of Infectious Diseases, Nanfang Hospital, Southern Medical University, 1838 Guangzhou Avenue North, Guangzhou, Guangdong China; 8 Department of Critical Care Medicine, Maoming People’s Hospital Affiliated to Southern Medical University, 101 Weimin Road, Maoming, 525000 Guangdong China

**Keywords:** Procalcitonin, C-reactive protein, Intensive care unit, Biomarker, Mortality, Predictor

## Abstract

**Background:**

It is not clear whether there are valuable inflammatory markers for prognosis judgment in the intensive care unit (ICU). We therefore conducted a multicenter, prospective, observational study to evaluate the prognostic role of inflammatory markers.

**Methods:**

The clinical and laboratory data of patients at admission, including C-reactive protein (CRP), were collected in four general ICUs from September 1, 2018, to August 1, 2019. Multivariate logistic regression was used to identify factors independently associated with nonsurvival. The area under the receiver operating characteristic curve (AUC-ROC), net reclassification improvement (NRI), and integrated discrimination improvement (IDI) were used to evaluate the effect size of different factors in predicting mortality during ICU stay. 3 -knots were used to assess whether alternative cut points for these biomarkers were more appropriate.

**Results:**

A total of 813 patients were recruited, among whom 121 patients (14.88%) died during the ICU stay. The AUC-ROC values of PCT and CRP for discriminating ICU mortality were 0.696 (95% confidence interval [CI], 0.650–0.743) and 0.684 (95% CI, 0.633–0.735), respectively. In the multivariable analysis, only APACHE II score (odds ratio, 1.166; 95% CI, 1.129–1.203; *P* = 0.000) and CRP concentration > 62.8 mg/L (odds ratio, 2.145; 95% CI, 1.343–3.427; *P* = 0.001), were significantly associated with an increased risk of ICU mortality. Moreover, the combination of APACHE II score and CRP > 62.8 mg/L significantly improved risk reclassification over the APACHE II score alone, with NRI (0.556) and IDI (0.013). Restricted cubic spline analysis confirmed that CRP concentration > 62.8 mg/L was the optimal cut-off value for differentiating between surviving and nonsurviving patients.

**Conclusion:**

CRP markedly improved risk reclassification for prognosis prediction.

## Background

Patients admitted to the ICU suffer from critical illness or injury and are at a high risk of dying. ICU mortality rates differ widely depending on the underlying disease process, with death rates as low as 1 in 20 for patients admitted following elective surgery and as high as 1 in 4 for patients with respiratory diseases [1]. The risk of death can be approximated by evaluating the severity of a patient’s illness as determined by important pathophysiological, clinical, and demographic determinants. In clinical practice, estimates of mortality risk can be useful in resource allocation, in determining appropriate levels of care, and even in discussions with patients and their families about expected outcomes.

The use of clinical risk scores, such as the Acute Physiology and Chronic Health Evaluation II (APACHE II) score or the Sequential Organ Failure Assessment (SOFA) score [2, 3], despite their considerable prognostic accuracy for ICU mortality, is partly also limited by practicality issues. For instance, certain disease states or conditions may generate very high severity scores, even though they do not generally result in high mortality. These are usually conditions associated with a high degree of physiological derangement but which are either self-limiting or can be managed to return towards normal relatively quickly. Classically, this arises with diabetic ketoacidosis but might also occur in patients admitted to ICU after surgery while still under the effects of general anesthesia [4]. Due to this uncertainty and drawback, physicians are often interested in the use of newly or clinically available predictive biomarkers that are objectively, rapidly, cost-effectively measurable, respond to clinical recovery, and add relevant, reliable, and real-time information [5].

As one of the major contributors for the all-cause mortality, systematic inflammatory response (SIR) is the common pathophysiological reaction in the critically ill patients [6, 7]. Markers of the SIR syndrome (SIRS), including CRP and PCT, as well as white blood cell count (WBC) have been shown to be prognostic of survival in patients in a variety of cancers [8–13]. However, the relationships between early CRP, PCT and WBC count at ICU admission and the mortality of severe patients have not been fully validated. We therefore conducted a multicenter, prospective, observational study to examine the possible independent relationships between the blood concentrations of the abovementioned inflammatory markers at ICU admission and ICU mortality in critically ill adults. The ability of the independent inflammatory markers for mortality prediction was further assessed.

## Methods

### Study design and setting

The study was conducted from September 1, 2018, to August 1, 2019, in four general ICUs of tertiary care hospitals in the Guangdong Province, China, which were multidisciplinary ICUs admitting patients from all medical areas with a specialty in surgery, including cardiothoracic surgery. When admitted into the ICU, patients were assessed for inclusion in the study. The inclusion criteria were: 1) length of ICU stay more than 24 h; 2) age over 15 years old; 3) informed consent signed. The exclusion criteria included any of the following: length of stay (LOS) in the ICU < 24 h; patients with thyroid tumors (e.g., thyroid adenoma or thyroid carcinoma); and inability to provide informed consent or unavailability of a proxy for informed consent. The primary endpoint was all-cause ICU mortality. The protocol was approved by the Institutional Ethics Committee of each participating center and was performed according to the ethical standards of the Declaration of Helsinki. Written informed consent was obtained from each patient or their legal surrogates. The study was registered at *http://www.chictr.org.cn/showproj.aspx?proj=29522* (ChiCTR1800017806).

### Laboratory measurements

All of the biomarkers were measured in a central laboratory, and all of the samples were labeled using study identification numbers without personal identifiers or clinical conditions. PCT was measured by Elecsys BRAHMS PCT (Roche Diagnostics GmbH, Germany; normal range, ≤0.05 μg/L). CRP was measured by an immunoenzyme analyzer (Hitachi 917, Tokyo, Japan; normal range, ≤5 mg/L). WBC counts were measured using an XE4000i automatic hemocyte analyzer. Blood samples for the purpose of study were collected only within 1 h after ICU admission, and clinicians decided the time and frequency of testing according to the actual clinical situation during the ICU stay.

### Data collection

In addition to PCT and CRP concentrations and WBC count, we also collected the demographic and clinical characteristics of each patient, including sex, age, treatment, preexisting chronic conditions, sepsis, Charlson score, source of admission, SOFA score, APACHE II score, and LOS in the ICU. ICU mortality data were collected by reviewing medical records in the in-hospital patient data management system. Sepsis was diagnosed according to the Surviving Sepsis Guidelines [4].

### Statistical analysis

Continuous variables are expressed as the median (IQR) and were compared with the Mann-Whitney U test; categorical variables are expressed as numbers (%) and were compared by the χ^2^ test or Fisher’s exact test between the survival and nonsurvival groups. All analyses were 2-tailed and conducted by SPSS for Windows (version 26.0; IBM, Chicago, IL, USA) and R Statistical Software (version 5.3.0). A *P* value < 0.05 was considered statistically significant.

Discrimination was evaluated using the area under the curve (AUROC) derived from the conventional receiver operating characteristic (ROC) curve. AUROC of > 0.5, > 0.6, > 0.70 or > 0.80 were considered poor, fair, satisfactory or good, respectively [12]. AUROCs, as a measure of classification accuracy, were further compared with or without CRP added to the APACHE II score using the nonparametric approach of DeLong and Clarke-Pearson [14]. Univariate and multivariate logistic regression analyses were used to detect factors independently associated with nonsurvival. The optimal cut-off values for individual biomarkers were determined using Youden’s index. To evaluate the utility of the biomarkers for risk classification, we determined the category-free net reclassification improvement (NRI) and the integrated discrimination improvement (IDI), as previously described [15, 16].

In consideration of the possibility that dichotomized cutoffs may not accurately capture the usefulness of PCT and CRP, sensitivity analysis was used to assess whether alternative cut points for these biomarkers were more appropriate. We used a restricted cubic spline function with 3 knots for PCT and CRP to allow nonlinearity as continuous predictors in a multivariable model [12, 17, 18].

## Results

### Baseline characteristics and outcomes of the patients

**Fig. 1:**
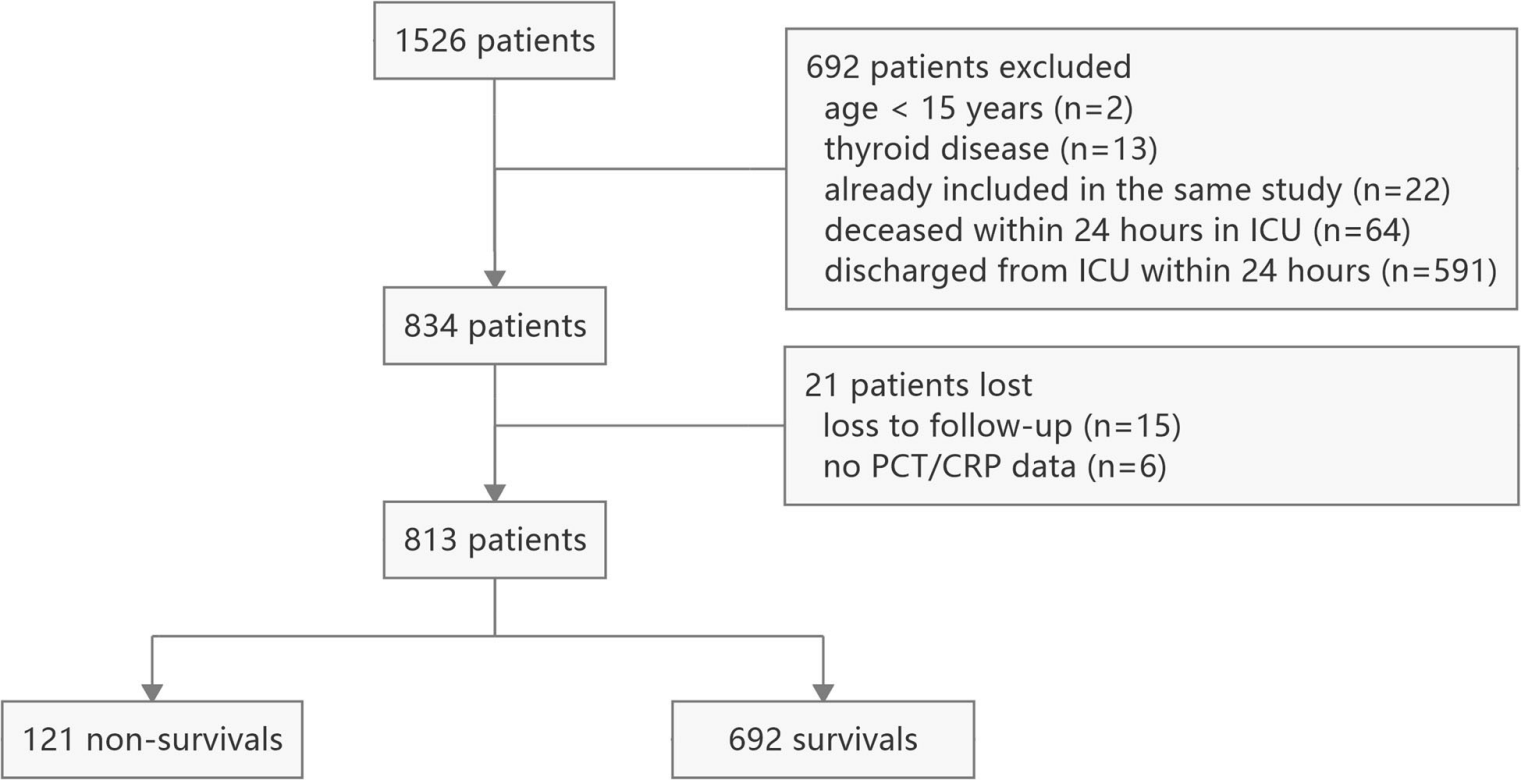
Flowchart showing inclusion and exclusion of patients for the study

Of the 1526 consecutive patients who were screened for inclusion in the study, 713 (46.7%) were excluded, and 813 patients were enrolled in the analyses (Fig. 1). The baseline characteristics and outcomes of the patients are summarized in Table 1. The serum concentrations of PCT and CRP were significantly higher in nonsurvivors than in survivors (PCT: 0.97 [0.23; 5.51] vs. 0.12 [0; 1.02] μg/L, *P* = 0.000; CRP 66.70 [12.80; 140.00] vs. 11.95 [2.25; 56.15] mg/L, *P* = 0.000). APACHE II score, SOFA score, age, source of admission, treatments, sepsis, preexisting clinical conditions including hypertension, COPD, coronary disease, diabetes mellitus and chronic heart failure also differed significantly between the two groups. The median days of LOS in the ICU were 6 and 3 in the survival and nonsurvival groups, respectively (Table 1).

**Table 1:** Baseline characteristics and outcome

Characteristics	Non-survival (*n* = 121)	Survival (*n* = 692)	*P* value
Demographic variables			
Male, n (%)	69 (57.02)	376 (54.34)	0.584
Age (years), median (IQR)	65 (52–76)	55 (43.5–67)	< 0.001
Preexisting clinical conditions			
Hypertension, n (%)	48 (39.67)	200 (28.90)	0.015
COPD, n (%)	12 (9.92)	23 (3.32)	0.002
Coronary disease, n (%)	28 (23.14)	54 (7.80)	< 0.001
Diabetes mellitus, n (%)	25 (20.66)	86 (12.43)	0.016
Malignant tumor, n (%)	23 (18.85)	128 (18.50)	0.894
Chronic heart failure, n (%)	10 (8.26)	22 (3.18)	0.011
Chronic liver disease, n (%)	4 (3.31)	25 (3.61)	0.867
Charlson score, median (IQR)	2 (1–3)	2 (1–2)	0.089
Source of Admission			
Surgery, n (%)	58 (47.93)	522 (75.43)	< 0.001
Internal medicine, n (%)	30 (24.79)	44 (6.36)	0.002
Emergency, n (%)	34 (28.10)	127 (18.35)	< 0.001
Treatment			
Mechanical ventilation, n (%)	97 (80.17)	465 (67.20)	0.005
RRT, n (%)	28 (23.14)	37 (5.35)	< 0.001
Use of corticosteroid, n (%)	32 (26.45)	148 (21.39)	0.217
Use of norepinephrine, n (%)	63 (51.64)	78 (11.40)	< 0.001
Inflammatory biomarkers, median (IQR)			
PCT (μg/L)	0.97 (0.23–5.51)	0.12 (0–1.02)	< 0.001
CRP (mg/L)	66.70 (12.80–140.00)	11.95 (2.25–56.15)	< 0.001
WBC (× 10^9^/L)	13.420 (9.070–17.810)	11.765 (8.395–15.645)	0.017
APACHEIIscore, median (IQR)	23 (18–29)	14 (9–19)	< 0.001
SOFA score, median (IQR)	8 (5–11)	2 (0–5)	< 0.001
Sepsis, n (%)	82 (67.77)	223 (32.23)	< 0.001
Outcome			
LOS of ICU (days), median (IQR)	6 (3–12)	3 (2–8)	< 0.001

*APACHEII* Acute Physiology and Chronic Health Evaluation II, *COPD* chronic obstructive pulmonary disease, *CRP* C-reactive protein, *ICU* intensive care unit, *IQR* interquartile range, *LOS* Length of stay, *PCT* procalcitonin, *RRT* Renal replacement therapy, *SOFA* Sequential Organ Failure Assessment, *WBC*, white blood count

### Risk factor analyses

In the univariate analyses, elevations in PCT concentrations (odds ratio [OR]: 1.010 [1.003; 1.018], *P* = 0.004) and CRP concentrations (OR: 1.008 [1.006; 1.011], *P* = 0.000) were associated with an increased risk of ICU mortality. Additionally, APACHE II score, age, sepsis, hypertension, diabetes mellitus, COPD, coronary disease, mechanical ventilation, RRT, use of norepinephrine and chronic heart failure also had this association, whereas WBC was not determined to be a risk factor (Table 2).

**Table 2:** Predictive characteristics of admission markers for intensive care unit mortality

Markers	Univariate Analysis				Multivariate Analysis			
	Cutoff	AUC (95% CI)	OR (95% CI)	P value	Cutoff	AUC (95% CI)	OR (95% CI)	P value
APACHEII score	20	0.816 (0.777–0.854)	1.174 (1.139–1.210)	< 0.0001	0.168^a^	0.823 (0.785–0.861)	1.163 (1.127–1.199)	< 0.0001
CRP (mg/L)	62.83	0.684 (0.633–0.735)	1.008 (1.006–1.011)	0.0037			2.145 (1.343–3.427)	0.003
PCT (μg/L)	0.33	0.696 (0.650–0.743)	1.010 (1.003–1.018)	0.0039				0.045
WBC (×10^9^/L)	16.23	0.568 (0.509–0.628)	1.013 (0.993–1.033)	0.1977				0.124

*APACHEII* Acute Physiology and Chronic Health Evaluation II, *AUC* Area under the receiver operating characteristic curve, *CRP* C-reactive Protein, *OR* odds ratio, *PCT* Procalcitonin, *WBC* White blood cell count

^a^ Cutoff point of the marker panels were the predicted probabilities corresponding to the best Youden’s index generated from the multiple logistic regression model

### ROC analyses

ROC analysis revealed that, as continuous predictors, none of the inflammatory markers, including PCT and CRP concentrations and WBC count at ICU admission, were considered satisfactory in discriminating between survival and nonsurvival. The AUC-ROC value of PCT for discriminating ICU mortality was 0.696 (95% CI: 0.650–0.743), that of CRP was 0.684 (95% CI: 0.633–0.735), and that of WBC was 0.568 (95% CI: 0.509–0.628). The same results were observed in the sepsis and nonsepsis subgroups (Supplementary Table 1). The optimal cut-off value of CRP for ICU mortality was 62.8 mg/L, and the sensitivity and specificity were 52.1% (95% CI, 42.8–61.2) and 76.7% (95% CI, 73.4–79.8), respectively. The positive and negative predictive values were 28.1% (95% CI, 22.3–34.5) and 90.2% (95% CI, 87.5–94.2), respectively.

**Fig. 2:**
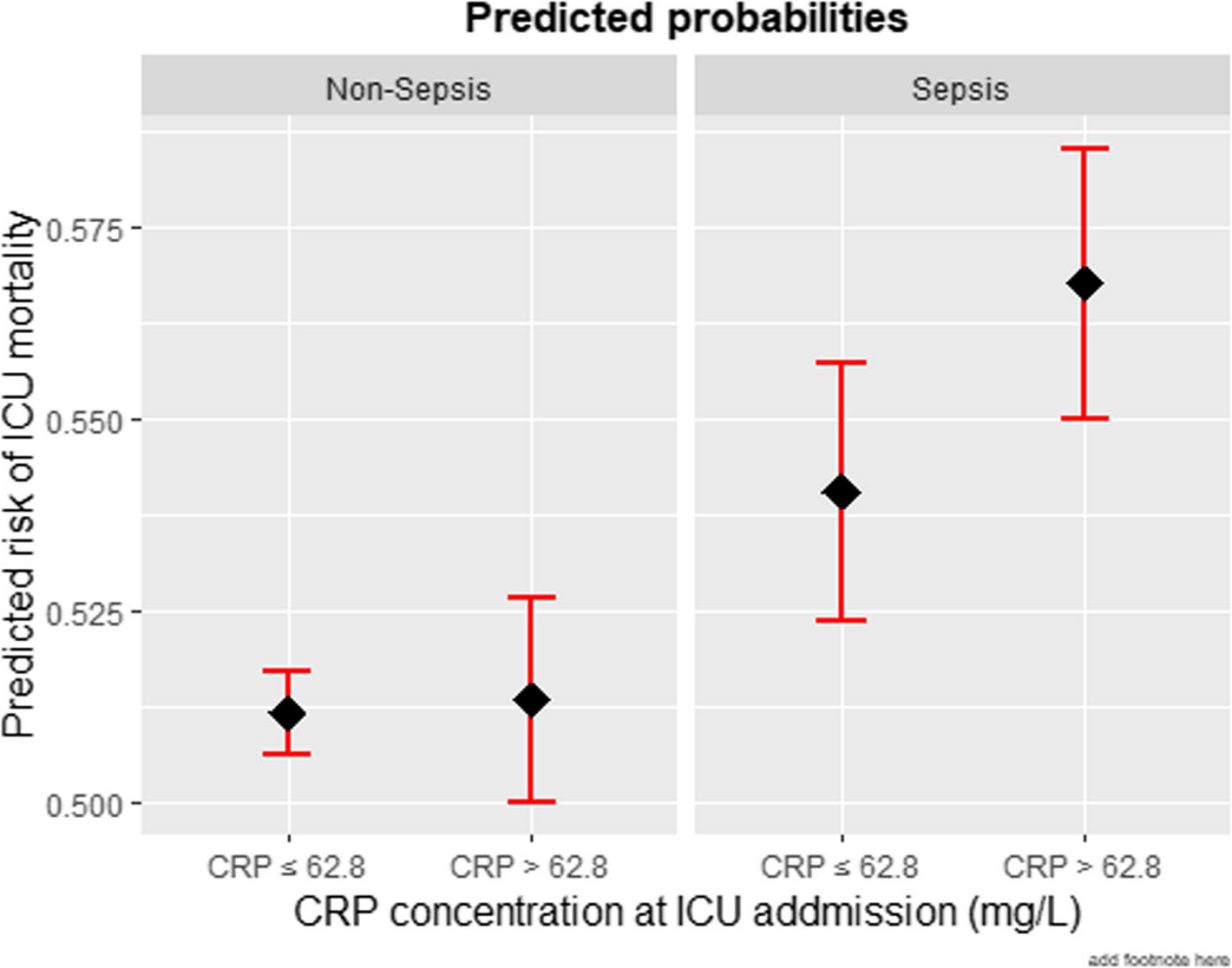
A high CRP concentration at admission in relation to predicted risk of ICU mortality stratified by sepsis and non-sepsis patients

**Fig. 3:**
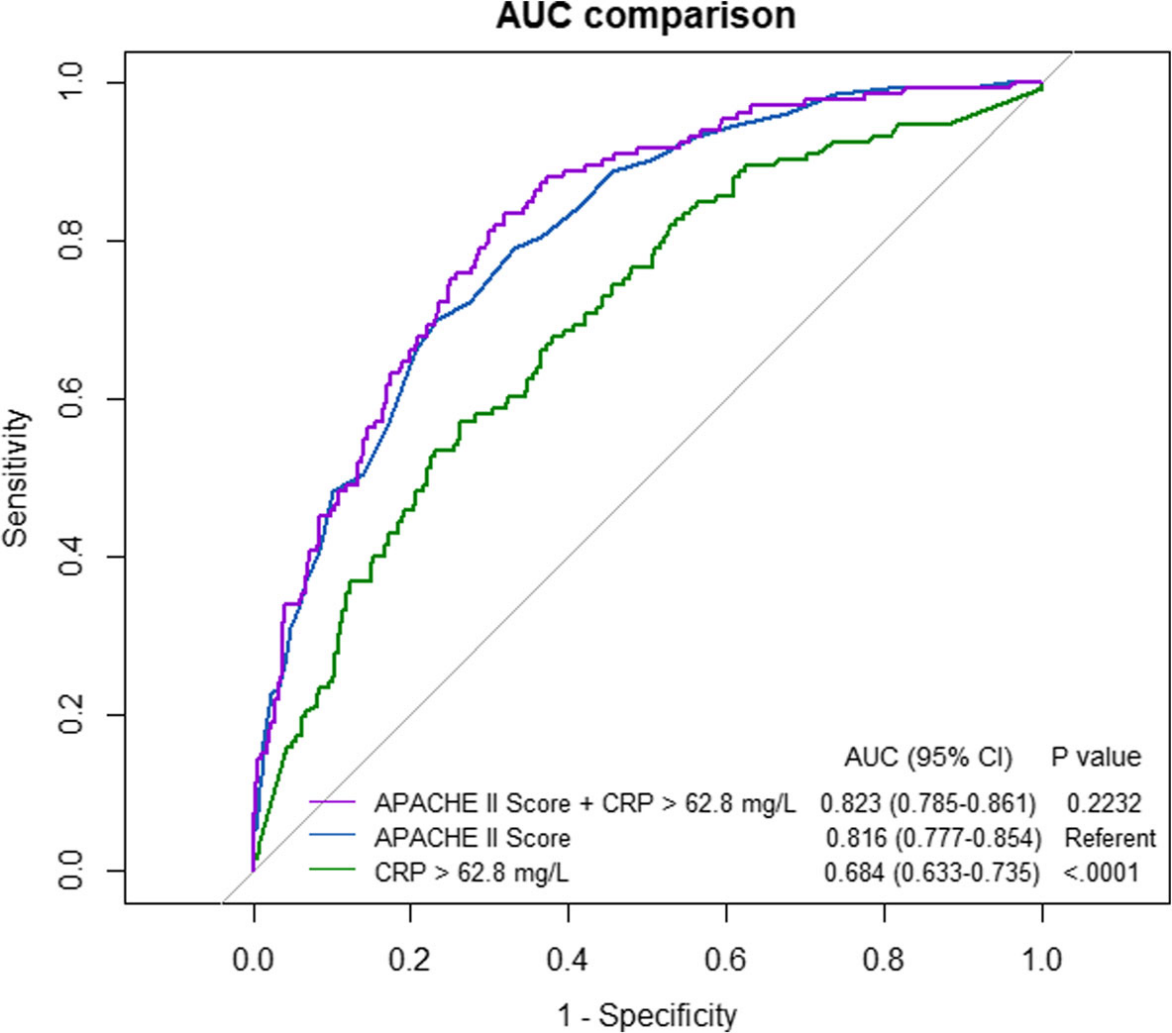
ROC analysis of CRP > 62.8 mg/L and APACHE II score biomarkers and their combinations for intensive care unit mortality prediction

In the multivariable analysis, only APACHE II score (odds ratio [OR], 1.166; 95% CI, 1.129–1.203; *P* = 0.000) and CRP concentration > 62.8 mg/L (OR, 2.145; 95% CI, 1.343–3.427; *P* = 0.001) were significantly associated with an increased risk of ICU mortality (Table 2), and this association did not seem to be significantly different between the sepsis and nonsepsis groups (Fig. 2). Moreover, adding the variable of CRP > 62.8 mg/L to the APACHE II score did not significantly increase the AUROC (Fig. 3).

### Effect of CRP on the risk reclassification of ICU mortality

Although CRP > 62.8 mg/L as a dichotomized variable did not significantly provide incremental value to the AUC of the APACHE II score, it markedly improved risk reclassification over the APACHE II score alone, displaying a category-free NRI of 0.556 (*P* < 0.0001) and an IDI of 0.013 (*P* = 0.0245) (Table 3). Our analysis suggested that the addition of CRP to the APACHE II score considerably improved the prediction accuracy of ICU mortality, mainly due to increasing the correct predicted probabilities for without events.

**Table 3:** NRI and IDI analyses for risk reclassification of ICU mortality

Models	Category-free NRI (95%CI)	*P* value	Category-free NRI (95%CI)				IDI (95% CI)	*P* value
			With Event	*P* value	Without Event	*P* value		
APACHE II score	Referent						Referent	
APACHE II score + CRP > 62.8 mg/L	0.556 (0.3705–0.7484)	<.0001	0.02 (0.0123–0.0324)	0.7851	0.53 (0.435–0.7371)	<.0001	0.013 (0.008–0.024)	0.0245

*APACHE II* Acute Physiology And Chronic Health Evaluation II, *CI* confidence interval, *CRP* C-reactive protein, *IDI* integrated discrimination improvement; *NRI* Net reclassification index

### Sensitivity analyses

**Fig. 4:**
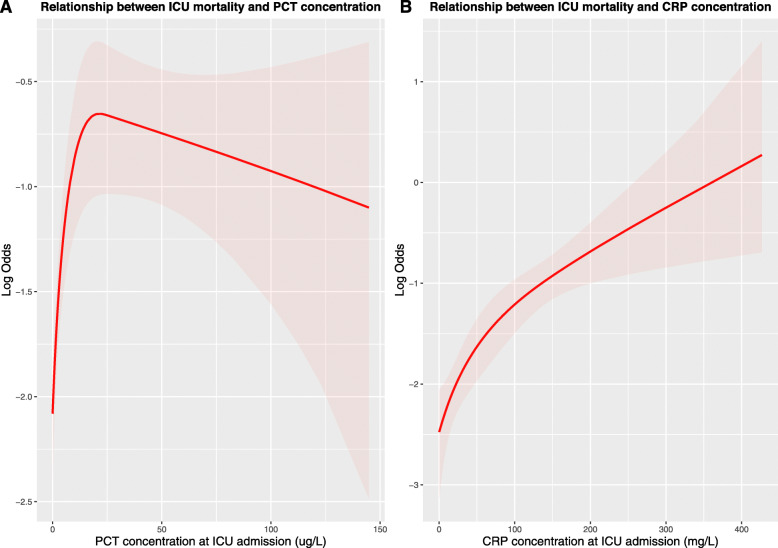
Relationship between risk of ICU mortality and PCT (A) and CRP (B) concentrations at ICU admission, allowing PCT and CRP as a nonlinear continuous predictor using a restricted cubic spline 3-knot function while adjusting for the APACHEIIscore, WBC, and CRP concentration. Shadow area show 95% CI

In the sensitivity analysis using a restricted cubic spline function for PCT, there was no obvious nonlinearity between PCT and the risk of ICU mortality (Fig. 4A). However, CRP concentration > 62.8 mg/L was confirmed to be the optimal cut-off value in differentiating between patients with survival and those without survival (Fig. 4B). The sensitivity and specificity of using CRP > 62.8 mg/L as a cut-off point to predict ICU mortality were 0.521 (95% CI, 0.43–0.72) and 0.767 (95% CI, 0.55–0.87), respectively. The positive and negative predictive values were 0.281 (95% CI, 0.214–0.516) and 0.902 (95% CI, 0.85–0.98), respectively.

## Discussion

In our study of critically ill patients, at ICU admission, the serum concentrations of PCT and CRP and WBC count were significantly lower in survivors than in nonsurvivors, which is similar to the findings of previous studies [12, 19]. As a continuous predictor, none of the inflammatory markers at ICU admission, including PCT concentrations, had sufficient discriminative ability for predicting ICU mortality. However, as a dichotomized variable, CRP > 62.8 mg/L at ICU admission was associated with an increased odds of ICU mortality regardless of whether the patient was septic or not. This association remained significant even after adjusting for the APACHE II score, WBC count, and PCT concentration. The findings have clinical implications that some clinically available inflammatory markers may be useful for helping clinicians assess the prognosis of patients in the ICU, which is worth further confirmation.

In clinical practice, PCT concentration is a well-established marker in septic patients [20–23]. However, this real-world study with a sizable sample did not provide evidence for the usefulness of PCT as a predictor of ICU mortality, and this negative association was not due to any nonlinear relationship between PCT and mortality. Although the PCT concentrations of nonsurvivors were significantly higher than those of survivors, the difference was not significant after adjusting for the APACHE II score, WBC count, and CRP concentration, and its AUC-ROC curve was not satisfactory. This result is not surprising since patients with different pathogens might have differently increased PCT values [24]. PCT elevation also occurs in nonsepsis states, such as postoperative conditions, cardiogenic shock or resuscitated cardiac arrest. In addition, PCT levels may be low in patients with viral infections, localized infections or early infections [25]. This negative finding may be due to the complicated reasons for ICU admission. On the other hand, even though the PCT concentration is increased in the serum of patients with bacterial infection, the half-life of PCT is short, and the maximum daily decrease under effective anti-infective therapy is 50% [26]. Therefore, with infection control, the PCT concentration decreases gradually, and the risk of death decreases accordingly. Otherwise, with the progression of infection, the PCT concentration continues to increase, and the risk of death also increases. This may be another reason why the discriminatory performance of PCT was poor in our population. This finding reminds us that more attention should be paid to the trend of PCT serum levels and not only one PCT value [20, 23, 27]. Of course, the time frame between two consecutive samples needs further investigation.

CRP has been used for many years [28–30]. However, as it is difficult to differentiate sepsis from other nonsepsis causes of SIR, its specificity has been challenged [31, 32]. In the study, the CRP concentrations of nonsurvivors were significantly higher than those of survivors. However, its AUC-ROC curve was not satisfactory. As a dichotomized variable, CRP > 62.8 mg/L at ICU admission was associated with an increased odds of ICU mortality regardless of whether the patient was septic or not. This may be related to the lack of specificity of CRP in both sepsis and nonsepsis patients [31]. The findings suggested that even inflammation without infection may still be associated with ICU mortality. The negative predictive value of CRP showed that patients with a low CRP (< 62.8 mg/L) at ICU admission had a low risk of ICU mortality. Therefore, in clinical practice, CRP may be useful for helping clinicians assess the prognosis of ICU patients.

In our study, APACHE II scores other than WBC count were independently associated with the outcome of nonsurvival. As the APACHE II score is a physiologically based system containing 12 physiological parameters, it is a useful prediction tool for hospital consequences, including mortality in critically ill patients [33]. Moreover, the APACHE II scoring system includes WBC count, and thus, pathophysiological changes after systemic insult could be illustrated comprehensively and systematically by the APACHE II scoring system. Therefore, this scoring system is thought to be superior to WBC count for the prediction of adverse outcomes [34].

Furthermore, we determined that the variable of CRP > 62.8 mg/L did not increase the prediction performance of the APACHE II score using the AUC-ROC comparison by the DeLong method. However, the AUC has recently been criticized for its insensitivity in model comparisons in which the baseline model has performed well [35]. Thus, 2 other measures have been proposed to capture the improvement in discrimination for nested models: integrated discrimination improvement and continuous net reclassification improvement. In the present study, we found that the dichotomized variable of CRP markedly improved risk reclassification over the APACHE II score, displaying a category-free NRI of 0.556 and an IDI of 0.013. On the basis of these results, we concluded that the APACHE II score plus CRP is more promising in terms of improving predictive value than the APACHE II score alone.

There are advantages in this study. To the best of our knowledge, this is the largest study exploring the prognostic value of PCT at ICU admission for patients with and without sepsis. Moreover, it is a multicenter prospective study that allows the retrieval of real-world data with potentially better data quality than retrospective designs, and our results may be generalizable to other centers that have different cases. However, there are some limitations in the study. First, many studies [36, 37] revealed that distinct groups of pathogens and different foci of infection determined different PCT serum concentration. In this study, the specific information about infections were not analyzed. It was due to the incomplete data regarding the pathogens and foci of infection. In addition to pathogens, there are many other contributing factors like the source of ICU admission, which showed difference in the constitution of the two groups. Second, CRP at ICU admission was associated with ICU mortality, and this association did not seem to be different between septic patients and nonseptic patients, but the sensitivity and specificity were not perfect. Third, many confounders may still exist and potentially determine bias. For example, some preexisting clinical conditions (i.e., hypertension, diabetes mellitus, coronary disease, diabetes mellitus) presented high proportions in the non-survival than in the survival group. Thus, further studies, such as those combining multiple biomarkers, are essential for improving the prediction performance.

PCT concentration and WBC count at ICU admission were inadequate in their predictive ability of ICU mortality. As a dichotomized variable, CRP > 62.8 mg/L at ICU admission was associated with an increased odds of ICU mortality regardless of whether the patient was septic or not. The negative predictive value of CRP showed that patients with a low CRP (< 62.8 mg/L) at ICU admission had a low risk of ICU mortality. Further studies, such as those combining multiple biomarkers, are essential for improving the prediction performance.

## Conclusions

For inflammatory biomarkers at ICU admission, the concentration of CRP, and not PCT or WBC, can be an independent risk factor for ICU mortality, and CRP can improve risk reclassification for prognosis prediction.

## Supplementary Information

**Additional file 1: Supplementary Table 1** Area under the ROC curve for inflammatory markers and clinical scoring systems at ICU admission in discriminating ICU mortality.

**Additional file 2.**

## Data Availability

The datasets used and/or analyzed during the current study are available from the corresponding author on reasonable request.

## Abbreviations

APACHE II: Acute Physiology and Chronic Health Evaluation II
AUC: Area under receiver operating characteristic curve
CI: Confidence interval
CRP: C-reactive protein
ICU: Intensive care unit
IQR: Interquartile range
OR: Odds ratio
PCT: Procalcitonin
ROC: Receiver operating characteristic curve
SD: Standard deviation
SIR: Systematic inflammatory response
WBC: White blood cell
