# Supplementary material for: C-reactive protein concentration as a risk predictor of mortality in intensive care unit: a multicenter, prospective, observational study
Source: BMC Anesthesiol. 2020 Nov 23;20:292. doi: 10.1186/s12871-020-01207-3 (PMC7680994; doi:10.1186/s12871-020-01207-3)
Supplement: Supplementary file 1 — Additional file 1: Supplementary Table 1 Area under the ROC curve for inflammatory markers and clinical scoring systems at ICU admission in discriminating ICU mortality. [file 12871_2020_1207_MOESM1_ESM.docx]

Supplementary Table 1 Area under the ROC curve for inflammatory markers and clinical scoring systems at ICU admission in discriminating ICU mortality

| Variables | AUC (95% CI) | | |
| --- | --- | --- | --- |
|  | All patients | Non-septic | Septic |
| PCT (μg/L) | 0.696 (0.650-0.743) | 0.677 (0.594-0.760) | 0.572 (0.503-0.640) |
| CRP (mg/L) | 0.684 (0.633-0.735) | 0.604 (0.515-0.693) | 0.603 (0.533-0.673) |
| WBC (×10^9^/L) | 0.568 (0.509-0.628) | 0.680 (0.588-0.772) | 0.524 (0.451- 0.597) |
| APACHEII score | 0.816(0.777-0.854) | 0.813 (0.742-0.883) | 0.743 (0.683-0.804) |

APACHEII, Acute Physiology and Chronic Health Evaluation II; AUC, Area under the ROC Curve; ROC, Receiver Operating Characteristic curve; CRP, C-reactive Protein; PCT, Procalcitonin; SOFA, Sequential Organ Failure Assessment; WBC, White Blood Cell.
