# Supplementary material for: C-reactive protein concentration as a risk predictor of mortality in intensive care unit: a multicenter, prospective, observational study
Source: BMC Anesthesiol. 2020 Nov 23;20:292. doi: 10.1186/s12871-020-01207-3 (PMC7680994; doi:10.1186/s12871-020-01207-3)
Supplement: Supplementary file 2 — Additional file 2. [file 12871_2020_1207_MOESM2_ESM.docx]

Figure 1


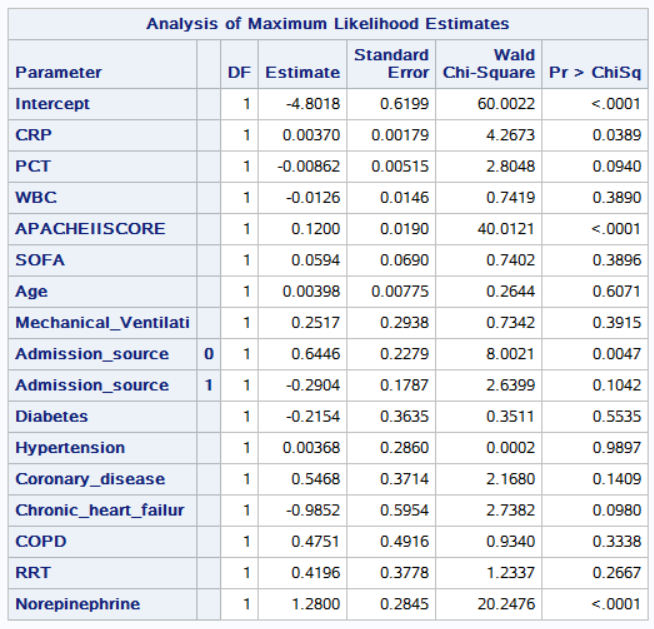


Table 1

| **Analysis of Maximum Likelihood Estimates** | | | | | | |
| --- | --- | --- | --- | --- | --- | --- |
| **Parameter** |  | **DF** | **Estimate** | **Standard Error** | **Wald Chi-Square** | **Pr > ChiSq** |
| **Intercept** |  | 1 | -4.8018 | 0.6199 | 60.0022 | <.0001 |
| **CRP** |  | 1 | 0.00370 | 0.00179 | 4.2673 | 0.0389 |
| **PCT** |  | 1 | -0.00862 | 0.00515 | 2.8048 | 0.0940 |
| **WBC** |  | 1 | -0.0126 | 0.0146 | 0.7419 | 0.3890 |
| **APACHEIISCORE** |  | 1 | 0.1200 | 0.0190 | 40.0121 | <.0001 |
| **SOFA** |  | 1 | 0.0594 | 0.0690 | 0.7402 | 0.3896 |
| **Age** |  | 1 | 0.00398 | 0.00775 | 0.2644 | 0.6071 |
| **Mechanical_Ventilati** |  | 1 | 0.2517 | 0.2938 | 0.7342 | 0.3915 |
| **Admission_source** | **0** | 1 | 0.6446 | 0.2279 | 8.0021 | 0.0047 |
| **Admission_source** | **1** | 1 | -0.2904 | 0.1787 | 2.6399 | 0.1042 |
| **Diabetes** |  | 1 | -0.2154 | 0.3635 | 0.3511 | 0.5535 |
| **Hypertension** |  | 1 | 0.00368 | 0.2860 | 0.0002 | 0.9897 |
| **Coronary_disease** |  | 1 | 0.5468 | 0.3714 | 2.1680 | 0.1409 |
| **Chronic_heart_failur** |  | 1 | -0.9852 | 0.5954 | 2.7382 | 0.0980 |
| **COPD** |  | 1 | 0.4751 | 0.4916 | 0.9340 | 0.3338 |
| **RRT** |  | 1 | 0.4196 | 0.3778 | 1.2337 | 0.2667 |
| **Norepinephrine** |  | 1 | 1.2800 | 0.2845 | 20.2476 | <.0001 |
